# Correction: Assabayev et al. Selenomethionine Mitigates Effects of *Nocardia cyriacigeorgica*-Induced Inflammation, Oxidative Stress, and Apoptosis in Bovine Mammary Epithelial Cells. *Int. J. Mol. Sci.* 2024, *25*, 10976

**DOI:** 10.3390/ijms27031383

**Published:** 2026-01-30

**Authors:** Talgat Assabayev, Jinge Han, Halihaxi Bahetijiang, Venera Abdrassilova, Muhammad Asfandyar Khan, Herman W. Barkema, Gang Liu, John P. Kastelic, Xueying Zhou, Bo Han

**Affiliations:** 1 College of Veterinary Medicine, China Agricultural University, Beijing 100193, China; talgata@cau.edu.cn (T.A.); halihaxi@cau.edu.cn (H.B.); asfand@cau.edu.cn (M.A.K.); gangliu@cau.edu.cn (G.L.); 2 College of Animal Science and Veterinary Medicine, Tianjin Agricultural University, Tianjin 300384, China; 2203020138@stu.tjau.edu.cn; 3 Department of Normal Physiology with Biophysics Course, Asfendiyarov Kazakh National Medical University, Almaty 050012, Kazakhstan; abdrasilova.v@kaznmu.kz; 4 Faculty of Veterinary Medicine, University of Calgary, Calgary, AB T2N 4N1, Canada; barkema@ucalgary.ca (H.W.B.); jpkastel@ucalgary.ca (J.P.K.)

In the original publication, there was a mistake in Figure 6 as published [1]. The authors confirmed that the error in Figure 6 originated during the initial drafting of the manuscript. The incorrect image was inadvertently inserted by the author and subsequently overlooked during the review process. The corrected Figure 6 appears below. The authors state that the scientific conclusions are unaffected. This correction was approved by the Academic Editor. The original publication has also been updated.

## Figures and Tables

**Figure 6:**
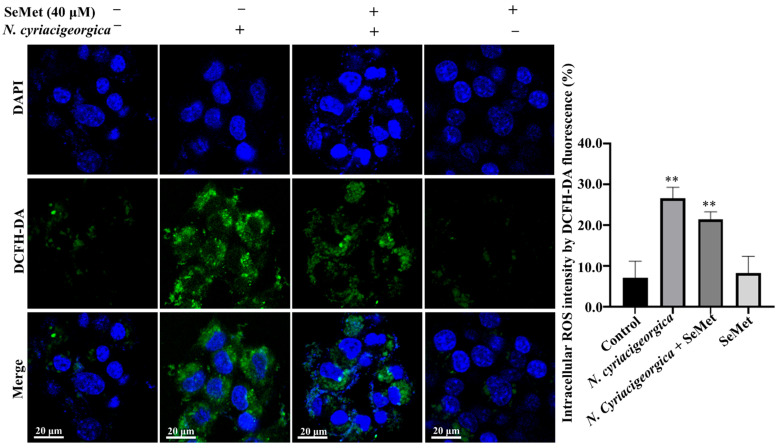
Effects of SeMet on *N. cyriacigeorgica*-induced increased in ROS concentrations in bMECs. In bMECs infected with *N. cyriacigeorgica*, ROS was increased (*p* < 0.01). However, in bMECs pretreated with 40 μM SeMet for 6 h before infection with *N. cyriacigeorgica* (MOI = 5:1), the ROS concentration was decreased (*p* < 0.01). In addition, ROS concentration of bMECs only pretreated with 40 μM SeMet for 6 h was not significantly different from the control. Data are means ± SD of three independent experiments. ** *p* < 0.01.
